# Broad-Scale Habitat Suitability and Fine-Scale Habitat Characterization of the Cerulean Warbler Using Species Distribution Modeling, Passive Acoustic Monitoring, LiDAR, and Satellite Remote Sensing

**DOI:** 10.3390/s26165152

**Published:** 2026-08-14

**Authors:** Adebola Esther Adeniji, Joseph Hupy, Bryan Pijanowski

**Affiliations:** 1 Department of Forestry and Natural Resources, Purdue University, West Lafayette, IN 47907, USA; bpijanow@purdue.edu; 2 Center for Global Soundscapes, Purdue University, West Lafayette, IN 47907, USA; 3 School of Aviation and Transportation Technology, Purdue University, West Lafayette, IN 47907, USA; jhupy@purdue.edu

**Keywords:** passive acoustic sensors, LiDAR, Sentinel 2, remote sensing, MaxEnt, Cerulean warbler, habitat structure

## Abstract

Understanding habitat requirements across spatial scales is important for conserving declining migratory species such as the Cerulean warbler. This study integrates data from several forms of remote sensing platforms: automated recording units, airborne LiDAR, and satellite remote sensing, to characterize the habitat of Cerulean warbler detection sites across central Indiana. We also modeled the suitable habitat of the species across the contiguous United States. Automated recording units were deployed across four forest types, and automated classification was used to derive species detections, which were manually validated. Structural variables derived within 25 m and 50 m buffers included LiDAR-based canopy height metrics, vertical vegetation distribution, foliage height diversity, and satellite-derived Enhanced Vegetation Index (EVI). Cerulean warbler detections were identified at 14 of the 57 acoustic sensor locations. Habitat characteristics at detection and non-detection sites were compared using univariate statistical tests and logistic regression models. Habitat associations varied with spatial scale. At 25 m, detection sites had significantly lower vegetation cover within the 5–10 m height stratum, which was also the highest-ranked candidate predictor, whereas EVI was the highest-ranked predictor at 50 m. At the broad scale, occurrence records from the Global Biodiversity Information Facility (GBIF) were integrated with climatic, topographic, land-cover, and anthropogenic variables within a MaxEnt modeling framework. The model showed moderate predictive performance and identified land cover as a key predictor of habitat suitability, with deciduous forests showing the highest probability of occurrence. This study highlights how multi-modal sensor data can be integrated for biodiversity monitoring and habitat assessment.

## 1. Introduction

Cerulean warblers (*Setophaga cerulea*) are Nearctic–Neotropical migratory songbirds and have been experiencing population decline in many parts of their range [1]. It is estimated that the species’ population declined by 3% annually from 1966 to 2000 [2]. The major causes of their decline are habitat loss and degradation across their full annual cycle [3]. Cerulean warblers are canopy-foraging insectivores that breed locally in mature and older deciduous forests and forage and nest higher in the canopy than most other species [4,5]. Due to its severe and steady decline in population levels, the Cerulean warbler has become a species of high conservation priority [6]. Understanding the potential distribution and habitat suitability of this species is crucial for informing conservation efforts that can guide habitat management.

Several studies have investigated fine-scale habitat selection and structural characteristics of the Cerulean warbler in localized portions of its breeding range, including the south-central United States [3], Southern West Virginia [7], southern Indiana [8], and northern Alabama [9]. While these studies provide valuable insights into local habitat requirements, their regional focus limits understanding of habitat suitability across broader spatial scales and does not capture variation across the species’ full breeding distribution. Most studies have relied on traditional field surveys such as point count [7,10], which has some limitations, such as observation bias from differences in skills between observers and might be biased towards rare, small, and cryptic species [11]. Studies have reported that it is difficult to sight female Cerulean warblers in the tree canopy [12]. Traditional field survey methods are resource intensive and can be invasive, making them limited in assessing their distributions. Most recently, these traditional field-based methods are now being complemented with sensing technologies such as passive acoustic monitoring, light detection and ranging (LiDAR), and electromagnetic-based satellite sensing [13].

Passive acoustic monitoring (PAM) is a method to survey wildlife by recording their vocalizations using passive acoustic sensors often referred to as Autonomous Recording Units (ARUs). PAM is a non-invasive observational method used to collect acoustic data to study ecological patterns without interfering with the environment [14]. It has been widely used in assessing biodiversity of animals, especially birds [15,16,17,18,19,20]. Advances in automated sound classifiers also have the potential to increase the likelihood of detecting rare species or cryptic individuals, especially canopy-dwelling birds that are difficult to observe due to the ease of long-duration recording processing [21]. Indeed, BirdNET, a freely available automated acoustic event classifier that uses a convolutional neural network algorithm, is being used to identify a large suite of bird species from passive acoustic monitoring recordings [22]. Habitat structural measures are now being developed using LiDAR, an active remote sensing technology, which is a more accurate and efficient tool used to describe the vertical vegetation structure and topography across large extents than a field survey [23,24]. LiDAR can allow for habitat analysis in areas not accessible for in-field assessments [25].

Despite increasing use of remote sensing and species distribution modeling in avian ecology, broad-scale habitat suitability modeling and fine-scale habitat characterization are often conducted independently for forest-dependent migratory songbirds [7,26]. Species distribution models provide valuable insights into the geographic distribution of suitable habitat across broad spatial extents, whereas fine-scale assessments quantify the local vegetation structure and habitat characteristics associated with species occurrence. Combining these complementary approaches can improve understanding of habitat requirements across different spatial scales and provide information that supports conservation planning for the Cerulean warbler. Most previous studies of Cerulean warbler habitat have relied on traditional field-based surveys, including point counts. In contrast, passive acoustic monitoring provides a non-invasive approach for detecting birds over extended sampling periods while reducing observer bias [14]. Coupling passive acoustic monitoring with airborne LiDAR and satellite remote sensing enables efficient characterization of forest structural and vegetation attributes associated with Cerulean warbler detections, providing a complementary approach for habitat assessment.

Therefore, this study evaluated Cerulean warbler habitat using complementary broad-scale and fine-scale approaches. At the broad scale, we developed a species distribution model using a maximum entropy (MaxEnt) approach to predict potential habitat suitability across the contiguous United States. MaxEnt is a general-purpose machine learning method whose performance has been evaluated as among the best among distribution modeling methods [27,28,29,30]. The broad-scale model incorporated climatic, topographic, and land-cover variables to identify major environmental drivers of habitat suitability across the contiguous United States. At fine scale, we integrated passive acoustic sensors with LiDAR-derived measurements of forest structure and Sentinel-2 vegetation indices to quantify habitat structure and vegetation characteristics associated with Cerulean warbler detection sites in west-central Indiana. Specifically, this study aimed to: (1) predict habitat suitability across the contiguous United States using MaxEnt, and (2) characterize local habitat structure at detection sites using passive acoustic monitoring integrated with LiDAR and Sentinel-2 remote sensing. Together, these complementary broad- and fine-scale analyses provide new insights into Cerulean warbler habitat and demonstrate the value of combining sensor-based monitoring, remote sensing, and species distribution modeling for conservation planning of forest-dependent songbirds.

## 2. Materials and Methods

### 2.1. Study Area

**Figure 1:**
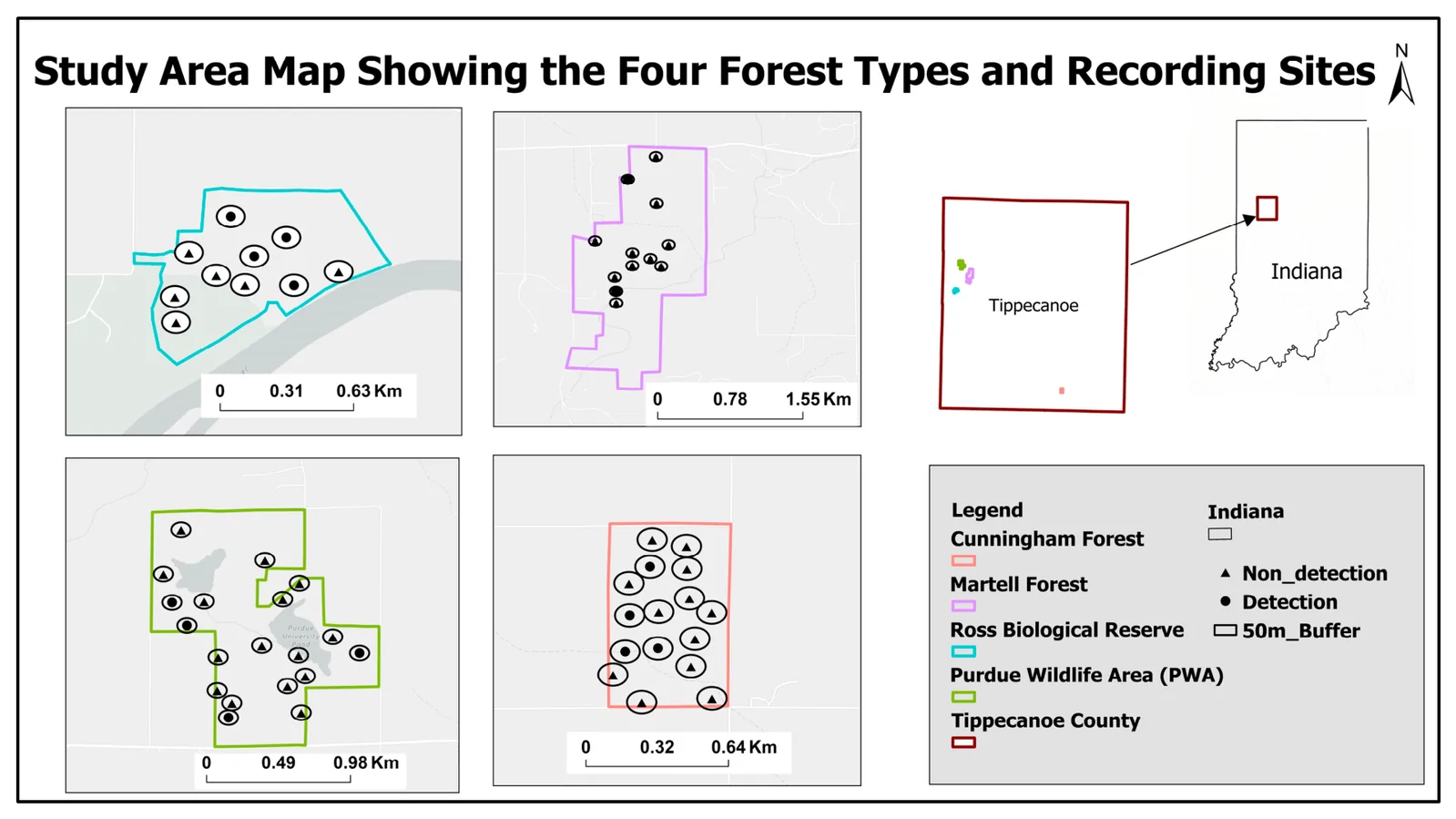
Study area map showing the location where the acoustic sensors were deployed.

The study was conducted at two spatial scales. Broad-scale habitat suitability was assessed across the contiguous United States. Fine-scale analysis was performed in west-central Indiana, United States (USA), where passive acoustic monitoring was implemented at 75 sensor locations distributed among four forest types: mature deciduous forest, mixed forest–grassland mosaic, mixed hardwood forest, and secondary managed forest (Figure 1). The study sites encompassed a range of forest habitat types in west-central Indiana: Ross Biological Reserve (67 acres; mature deciduous forest), Purdue Wildlife Area (290 acres; mature forest–grassland mosaic), Martell Forest (477 acres; secondary managed forest), and Cunningham Forest (80 acres; mixed hardwood forest). Purdue University manages all four sites.

### 2.2. Broad-Scale Habitat Modeling

- Occurrence data

Occurrence records of the Cerulean warbler (*Setophaga cerulea*) were obtained from the Global Biodiversity Information Facility (GBIF) for the year 2023 to represent the species’ distribution across the contiguous United States. The species arrives in its breeding range in early May and departs in August. Records were filtered to include only observations from May through August within the contiguous United States. Data cleaning procedures included removal of duplicate records, elimination of observations with missing or erroneous geographic coordinates, and verification of spatial accuracy. To reduce spatial sampling bias and spatial autocorrelation, occurrence records were spatially thinned using the spThin package with a minimum nearest-neighbor distance of 5 km, resulting in one occurrence per thinning distance. After filtering and thinning, a total of 1604 occurrence points were retained for modeling.

To reduce the influence of spatial sampling bias associated with occurrence records, a target-group background approach was implemented instead of randomly sampling background locations [31]. Breeding-season (May–August) occurrence records of North American wood warblers (Family: Parulidae), excluding the Cerulean warbler, were downloaded from the Global Biodiversity Information Facility (GBIF). Records were filtered to retain only observations with valid geographic coordinates within the contiguous United States, and duplicate records within the same raster cell were removed. The target-group records were used to generate background locations across the entire contiguous United States (CONUS), which is similar to the model calibration extent. A random subset of 10,000 background locations was selected for model calibration. Because the target-group records were collected using the same observation platform and survey methods as the focal species, they provide a spatial representation of observer sampling effort and reduce bias associated with uneven sampling intensity among spatial units [31].

- Environmental variable

Environmental variables consisted of bioclimatic data, elevation data, land cover, hydrological features, and measures of anthropogenic disturbance that are distributed at the continental scale. Nineteen bioclimatic variables were obtained from the WorldClim database (accessed on 21 September 2024) at a spatial resolution of 30 arc-seconds (approximately 1 km). To eliminate collinearity among bioclimatic variables, we excluded those with correlation coefficients exceeding 0.7, leaving 4 bioclimatic variables relevant to the Cerulean warbler: Bio7 (Temperature Annual Range), Bio10 (Mean Temperature of the Warmest Quarter), Bio15 (Precipitation Seasonality), and Bio18 (Precipitation of the Warmest Quarter). Variance inflation factor (VIF) analysis was performed on the retained continuous predictors, and all variables included in the final model had VIF values below 5.

Elevation data from the U.S. Geological Survey (USGS) 3D Elevation Program were used to derive slope (degree) and aspect. Aspect was transformed into northness (cosine of aspect) and eastness (sine of aspect) before modeling, because aspect is a circular variable (0° and 360° represent the same direction). Land cover data for the year 2023 were obtained from the USGS National Land Cover Database (NLCD). Distance to major rivers and roads was calculated in ArcGIS Pro (v. 3.5) using the Euclidean Distance tool with roads and rivers shapefiles obtained from the United States Census Bureau TIGER/Line dataset to represent hydrological features and human disturbance. Details on data sources are provided in Table 1.

All environmental layers were processed in ArcGIS Pro (v. 3.5) and R (v 4.4) to ensure a common coordinate reference system, spatial resolution, grid alignment, and geographic extent. The WorldClim bioclimatic variables (30 arc-seconds; ~1 km) were used as the reference grid. Continuous predictors, including elevation, slope, aspect, and Euclidean distance to roads and rivers, were reprojected and resampled to match the WorldClim grid using bilinear interpolation. The categorical National Land Cover Database (NLCD) layer was reprojected and aggregated to the 1 km modeling grid using a majority-rule approach, thereby preserving the dominant land-cover class within each model cell (Table 2). All predictor variables were aligned to a common raster geometry while maintaining the effective spatial resolution of the habitat suitability model at approximately 1 km, corresponding to the coarsest predictor dataset (WorldClim).

- Habitat Suitability Modeling

Species distribution model was developed using the maximum entropy algorithm implemented in MaxEnt version 3.4.4, a machine-learning method designed for presence-only data. Model tuning and evaluation were performed in R using the ENMeval package to choose the parameters that exhibit the greatest performance based on predictive power, relevance, and model complexity level [32,33,34]. Environmental predictors included the retained bioclimatic variable, elevation, northness and eastness, slope, distance to roads, distance to rivers, and the National Land Cover Database (NLCD) Table 2. Model complexity was optimized using the ENMeval package with spatial block cross-validation (4-fold cross-validation). Candidate models were evaluated using combinations of regularization multipliers ranging from 0.5 to 4.0 (increments of 0.5), and multiple feature class combinations (L, LQ, H, LQH, LQHP, and LQHPT, where L = linear, Q = quadratic, H = hinge, P = product, and T = threshold). To select the optimal model that best balanced predictive performance and model complexity, models were compared using Akaike’s Information Criterion corrected for small sample size (AICc), validation area under the receiver operating characteristic curve (AUC) [35,36], omission rate, and the difference between training and validation AUC.

Following model selection, the optimal feature class combination and regularization multiplier identified by ENMeval were used to fit the final MaxEnt model using all filtered occurrence records and the complete set of selected environmental predictors. The final model generated continuous habitat suitability predictions using the cloglog output format, which estimates the probability of species occurrence. Model performance can be improved by cloglog transformation by reducing the effects of sample selection bias, while maintaining the same AUC [37]. AUC values range from 0 to 1. AUC thresholds exceeding 0.9 typically indicate high predictive reliability, while values below 0.7 suggest limited model efficacy [38]. Habitat suitability was projected across the contiguous United States, and predictor importance was assessed using MaxEnt’s percent contribution. MaxEnt outputs are expressed as probability values ranging from 0 to 1, representing the relative suitability of habitat across the study area. Values approaching 1 indicate highly suitable conditions for the species, whereas values closer to 0 reflect low suitability [39,40]. The continuous suitability surface was imported into ArcGIS Pro (v. 3.5) and reprojected to the USA Contiguous Albers Equal Area Conic coordinate system for visualization and map production. All computationally intensive analyses, including environmental raster processing and MaxEnt model calibration and tuning, were performed on Purdue University’s Rosen Center for Advanced Computing (RCAC) high-performance computing cluster using R (version 4.5.2).

### 2.3. Fine-Scale Data and Analysis

- Passive acoustic monitoring and BirdNET analyzer

We conducted passive acoustic monitoring at 75 sensor deployment locations distributed across four forest types (mature deciduous forest, mixed forest–grassland mosaic, mixed hardwood forest, and secondary managed forest) in west-central Indiana to record and detect the presence of the Cerulean warbler in the forests in May 2025 (Figure 1). We deployed 5 acoustic sensors (Wildlife Acoustics© (Maynard, MA, USA), Song Meter, including versions SM4 and SM3) in each forest type every week for four weeks, with each deployment recording for three days, with a duty cycle of 1 min on and 9 min off. resulting in 432 recordings per sensor. However, we were unable to complete the fourth deployment in the secondary managed forest due to inaccessibility. To minimize spatial dependence among sampling locations, the Near tool in ArcGIS Pro (v 3.5) was used to calculate the distance between all sensor locations. Sensors located within 100 m of another sensor were excluded from the habitat analysis to avoid overlapping sampling areas and reduce the potential for spatial pseudoreplication. The 100 m threshold was selected as a minimum separation distance to reduce clustering while also retaining sufficient sampling locations for analysis. However, because the vocalization of Cerulean warbler may propagate beyond 100 m and nearby locations within the same forest reserve may share similar environmental conditions, this threshold was not assumed to guarantee complete acoustic or ecological independence. Following this filtering step, the final dataset consisted of 57 sensor locations separated by at least 100 m.

We processed data using the algorithm BirdNET analyzer version 2.1 [22], an automated deep-learning algorithm for avian sound identification. Analyses were conducted using default detection parameters (sensitivity = 1.0, overlap = 0) and incorporating the geographic coordinates of the study area to improve classification accuracy. To evaluate BirdNET classification performance for the Cerulean warbler, detections were stratified into 0.05 confidence intervals covering the full range of BirdNET confidence scores (0.05–0.10, 0.10–0.15, …, 0.90–0.95) [41,42]. Because the majority of detections occurred within the two lowest confidence intervals, 20 detections were randomly selected from each of these intervals (0.05–0.10 and 0.10–0.15) to provide greater representation of low-confidence classifications. Ten detections were randomly selected from each remaining confidence interval [41]. When a confidence interval contained fewer than the target number of detections, all available detections were included in the manual validation. The number of total detections, manually validated detections, false positives, true positives, and precision within each confidence interval is provided in Table A1.

**Figure A1:**
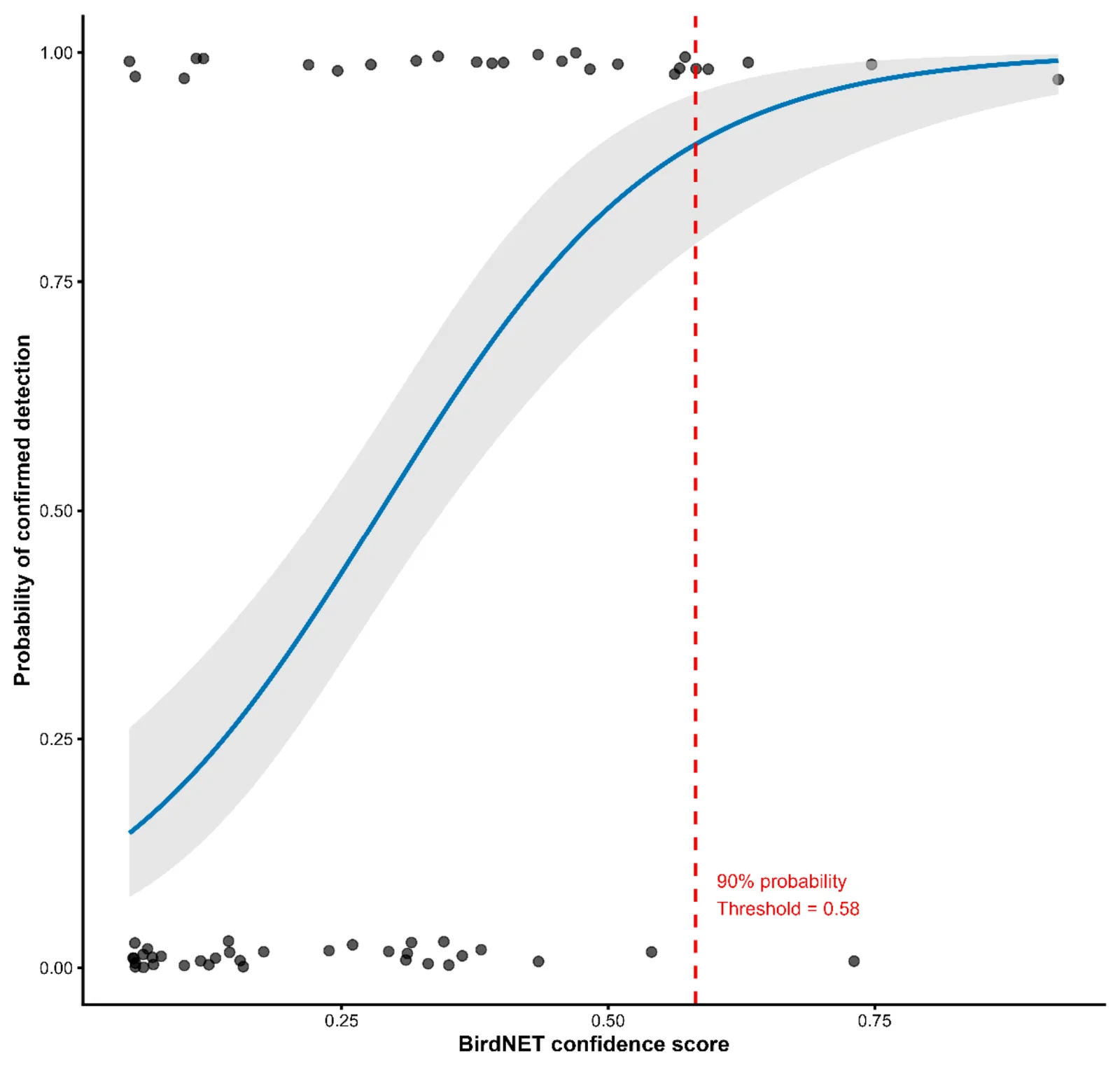
Logistic regression showing the relationship between BirdNET confidence score and the probability of a manually confirmed Cerulean warbler detection. The blue line represents the fitted logistic regression model, the gray shaded area indicates the 95% confidence interval, and points represent manually validated detections (1) and non-detections (0). The red dashed vertical line indicates the confidence threshold corresponding to a predicted 90% probability of a true Cerulean warbler detection (confidence score = 0.58).

**Figure A2:**
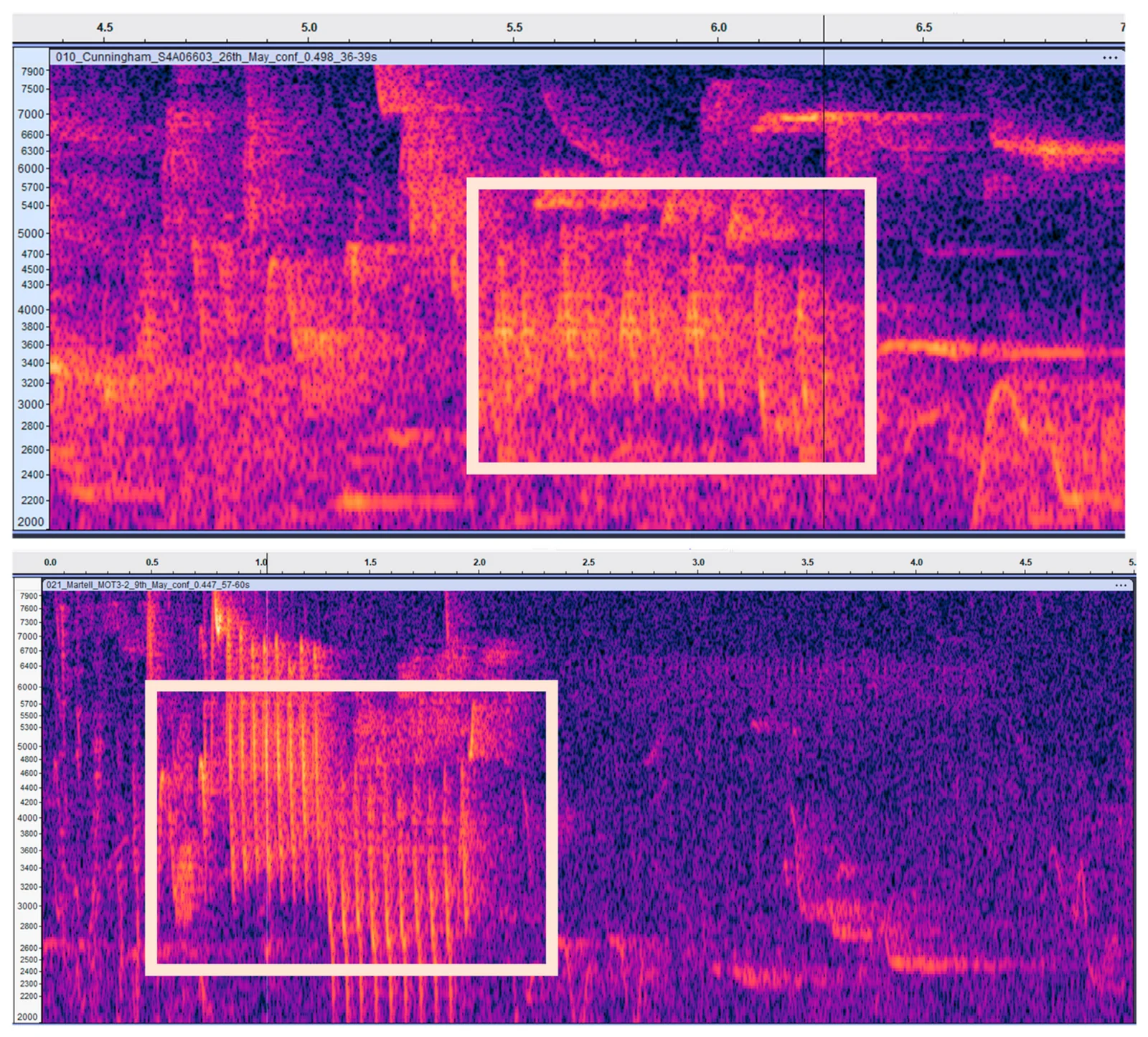
Representative spectrograms of a manually confirmed Cerulean warbler vocalization. The highlighted vocalization (Light orange box) was confirmed through manual review of the corresponding audio recording and is provided as an example of the validation process. Spectrogram colors represent relative acoustic signal intensity, with brighter orange/yellow colors indicating greater signal intensity and darker purple colors indicating lower intensity. Time (s) is displayed along the top of each spectrogram, and frequency (Hz) along the left axis. Spectrograms were generated and exported from Audacity.

Precision was calculated as the proportion of true positives of manually reviewed BirdNET detections of Cerulean warbler among identified predictions [41]. Precision was first summarized within each 0.05 confidence interval, and then a generalized linear model was fitted with a binomial distribution to generate a calibration curve, using BirdNET confidence values as the predictor variable and true/false positive classifications as the response variable [43]. Although the fitted logistic model estimated that a confidence score of 0.58 corresponded to a 90% precision (Figure A1), manual validation showed that a confidence threshold of 0.40 achieved similarly high observed precision while retaining a substantially larger number of validated detections and confirmed sensor locations. Because the primary objective was to minimize false positives while retaining sufficient ecological information, 0.40 represented the best balance between precision and data retention (Table A1). To verify site-level acoustic status, BirdNET detections exceeding the selected confidence threshold (0.40) were manually reviewed for each positive sensor location (Table A2). For sites with multiple detections, the highest-confidence recordings were examined. Manual validation consisted of listening to each extracted audio clip while simultaneously inspecting its spectrogram using Audacity (version 3.7.5) Figure A2. A site was considered confirmed when at least one recording contained a vocalization that was independently identified as Cerulean warbler.

- LiDAR and satellite remote sensing

LiDAR and satellite remote sensing datasets were used to quantify fine-scale habitat characteristics at Cerulean warbler detection sites. Sentinel-2 surface reflectance imagery was obtained from the harmonized Sentinel-2 Level-2A collection (COPERNICUS/S2_SR_HARMONIZED) in Google Earth Engine. Images intersecting the study locations were filtered to May 2025 and to scenes with <30% scene-level cloud cover. Surface reflectance values for the blue (B2), red (B4), and near-infrared (B8) bands were scaled by 0.0001 before calculating the Enhanced Vegetation Index (EVI). The B2, B4, and B8 bands have a spatial resolution of 10 m in this product. EVI was calculated for each image, and a monthly median composite was generated.

EVI was calculated as:

$$EVI=2.5\times \frac{NIR\;-\;Red}{NIR+6\times Red-7.5\times Blue+1}$$

where the near-infrared (NIR), red, and blue reflectance corresponded to Sentinel-2 bands B8 (842 nm), B4 (665 nm), and B2 (490 nm), respectively.

Mean EVI values were then extracted within 50 m buffers around each sensor location. Airborne LiDAR data acquired in 2018 were obtained from the Indiana Statewide LiDAR dataset (https://lidar.digitalforestry.org/ accessed on 4 December 2025), which follows Quality Level 2 (QL2) standards. Point density was approximately 3.2 points m^−2^ at Cunningham and 4.3 points m^−2^ at Martell, PWA, and Ross. LiDAR processing was conducted in R using lidR version 4.2.2. The dataset has a hydro-flattened bare-earth digital terrain model with an original pixel resolution of 2.5 ft (~0.76 m) across most of Indiana and meets the USGS 3DEP vertical. LiDAR point heights were normalized to height above ground using lidR::normalize_height(), and the corresponding bare-earth digital elevation model (DEM), and normalized heights were converted from US survey feet to meters. Returns with negative normalized heights were excluded. The source point clouds contained first through fifth returns, and no filtering by return number was applied. Source LAS classifications were dominated by processed but unclassified (class 1) and ground (class 2) points, with small proportions of other classifications, including low noise (class 7), water (class 9), rail (class 10), bridge deck (class 17), and high noise (class 18), depending on the reserve. Points classified as low noise (class 7) or high noise (class 18) were excluded before calculation of vegetation metrics.

LiDAR returns were extracted within 50 m buffers around each acoustic sensor using lidR::clip_roi(). Mean, maximum, standard deviation, and the 25th, 50th, 75th, and 95th percentiles of normalized height were calculated using lidR::stdmetrics_z(). Vegetation-cover metrics were calculated as the number of retained returns within each specified height interval divided by the total number of retained returns with normalized height ≥0 m within the buffer. Specifically, cover was calculated for 0–5 m, >5–10 m, >10–20 m, and >20 m strata, as well as the proportions of returns > 2 m and >5 m. Foliage-height diversity was calculated using the Shannon diversity index based on the proportions of retained returns within 5 m vertical bins.

To evaluate whether fine-scale habitat associations were sensitive to the spatial extent used to characterize vegetation structure, the analysis was repeated using a 25 m-radius buffer around each sensor. The 50 m radius was retained as the primary analysis, whereas the 25 m analysis was treated as a sensitivity analysis. A supplementary assessment of potential forest disturbance during the intervening period was conducted using the Hansen Global Forest Change dataset because the airborne LiDAR data were acquired in 2018, seven years before the 2025 acoustic surveys. Mapped tree-cover loss from 2019–2025 was quantified within 50 m buffers surrounding each of the 57 unique sensor locations. The proportion of each buffer affected by mapped tree-cover loss was calculated to identify locations potentially affected by major canopy disturbance after LiDAR acquisition.

### 2.4. Data Analysis

Habitat variables extracted within 50 m buffers surrounding each sensor location were compared between Cerulean warbler detection and non-detection sites. To evaluate the sensitivity of fine-scale habitat associations to the spatial scale of habitat characterization, the analyses were repeated using a 25 m radius as a secondary sensitivity analysis. Before analysis, the assumptions of normality and homogeneity of variances were evaluated using the Shapiro–Wilk test and Levene’s test, respectively. Variables satisfying these assumptions were compared using two-sample Student’s *t*-tests, whereas variables violating one or both assumptions were compared using the Mann–Whitney U test. These analyses were considered exploratory because they were intended to identify candidate predictors for subsequent logistic regression. Effect sizes were reported as Cohen’s d for t-tests and rank-biserial correlation (r) for Mann–Whitney U tests. Variables analyzed using Mann–Whitney U tests are summarized using medians and interquartile ranges (IQR).

**Figure A3:**
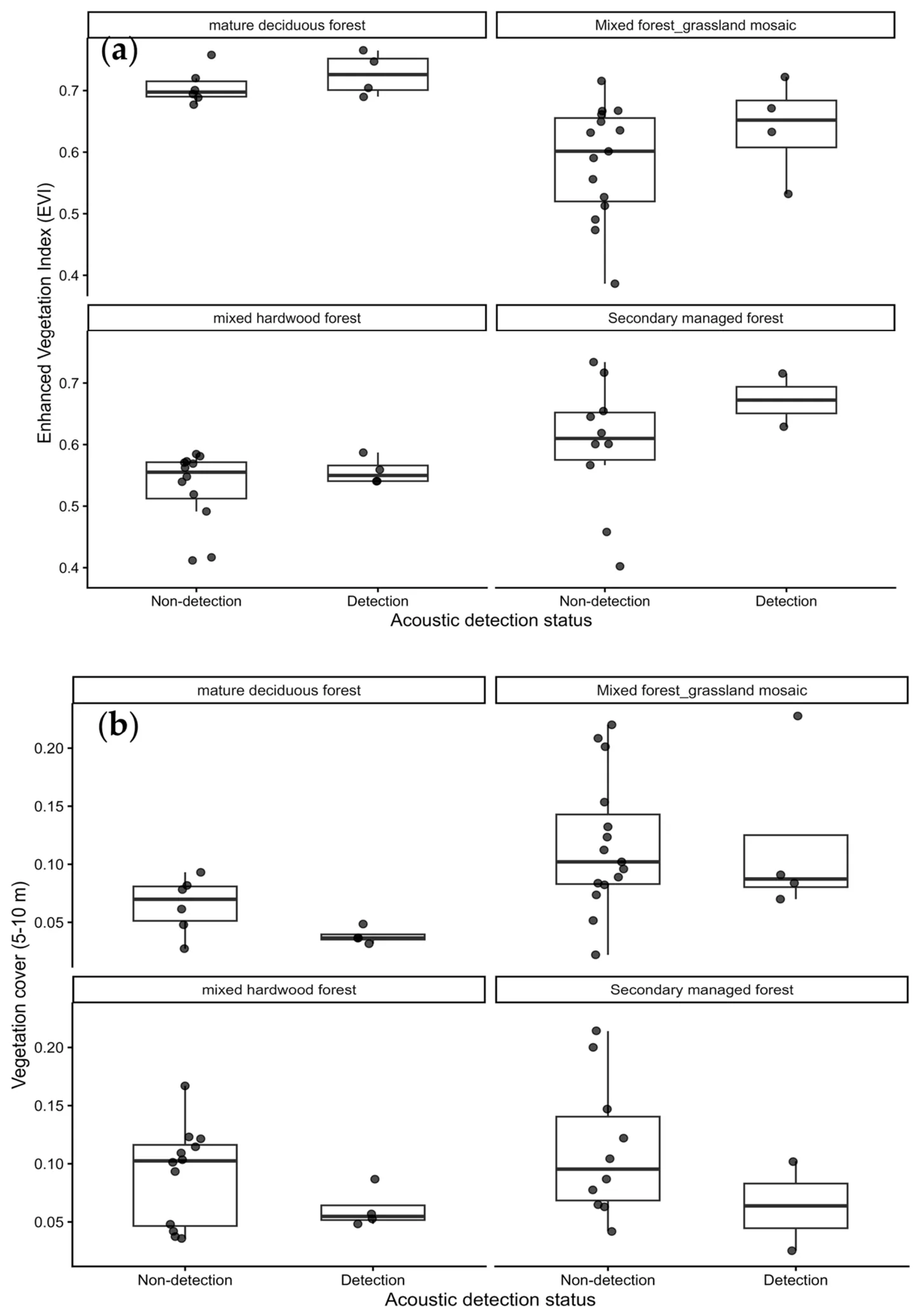
Distribution of the highest-ranked fine-scale habitat predictors across the four study reserves by Cerulean warbler acoustic detection status. (**a**) Enhanced Vegetation Index (EVI) is shown for the 50 m buffer analysis, and (**b**) Vegetation cover within the 5–10 m height stratum is shown for the 25 m buffer analysis. Boxes represent the interquartile range with the median indicated by the center line, and points represent individual sensor locations.

To identify habitat variables associated with Cerulean warbler detection, a series of candidate generalized linear models (GLMs) with a binomial error distribution and logit link function were fitted using detection status (detection = 1, non-detection = 0) as the response variable and selected habitat variables as predictors [44,45]. Separate candidate generalized linear models were constructed for habitat variables representing canopy height (z_mean), lower-canopy height (z_p25), midstory vegetation cover (cover_5_10), subcanopy vegetation cover (cover_10_20), and vegetation productivity (EVI). An intercept-only null model was also fitted to provide a baseline for comparison. Candidate variables were selected after evaluating pairwise correlations and variance inflation factors to retain predictors that were not highly correlated (|r| < 0.70; VIF < 3), thereby minimizing redundancy among habitat variables [44]. Because the number of Cerulean warbler detection sites was limited, each habitat variable was evaluated in a separate logistic regression model, and candidate models were compared using Akaike’s Information Criterion [46]. Models were compared using Akaike’s Information Criterion corrected for small sample size (AICc), and the model with the lowest AICc was considered the best-supported model. Model coefficients were used to evaluate the direction and strength of associations between habitat variables and the probability of Cerulean warbler detection. The highest-ranked logistic regression model at each spatial scale was additionally fitted using Firth-penalized logistic regression to reduce potential small-sample bias because only 14 sensor locations contained confirmed Cerulean warbler detections. Regression coefficients, profile penalized likelihood 95% confidence intervals, and odds ratios are reported from the penalized model. To evaluate whether the focal habitat associations were influenced by clustering among the four study reserves were influenced by clustering among the four study reserves, detection and non-detection locations were summarized by reserve (Table A3), and the distribution of 5–10 m vegetation cover was examined by reserve and detection status (Figure A3). Residual spatial autocorrelation from the focal logistic regression model was evaluated using Moran’s I based on a four-nearest-neighbor spatial weights matrix constructed from sensor coordinates, with significance assessed using 999 Monte Carlo permutations. A leave-one-reserve-out sensitivity analysis was also conducted by sequentially excluding each reserve and refitting the Firth-penalized logistic regression (Table A4).

## 3. Results

### 3.1. Broad-Scale Habitat Suitability Model of Cerulean Warbler

- Model Evaluation

The optimal MaxEnt model used LQHPT feature classes with a regularization multiplier of 1.0, selected based on the lowest AICc (47,837.91; ΔAICc = 0; AIC weight = 0.997342). The model achieved a training AUC of 0.831 and a mean validation AUC of 0.760 under spatial block cross-validation. Validation performance varied among the four spatial blocks, with AUC values ranging from 0.703 to 0.835 and continuous Boyce index (CBI) values ranging from 0.736 to 0.984 (Table 3). The mean fold-specific training–validation AUC difference was 0.081 ± 0.060, indicating some reduction in predictive performance when the model was evaluated on spatially withheld data. The mean 10% training omission rate was 0.231, whereas the minimum training presence omission rate was 0.002. Details of species distribution model validation and evaluation metrics are provided in the Supplementary Materials, Tables S1 and S2. Thus, the MaxEnt model is suitable for assessing the habitat suitability of Cerulean warbler across the contiguous United States.

- Variable contribution and importance

**Figure A4:**
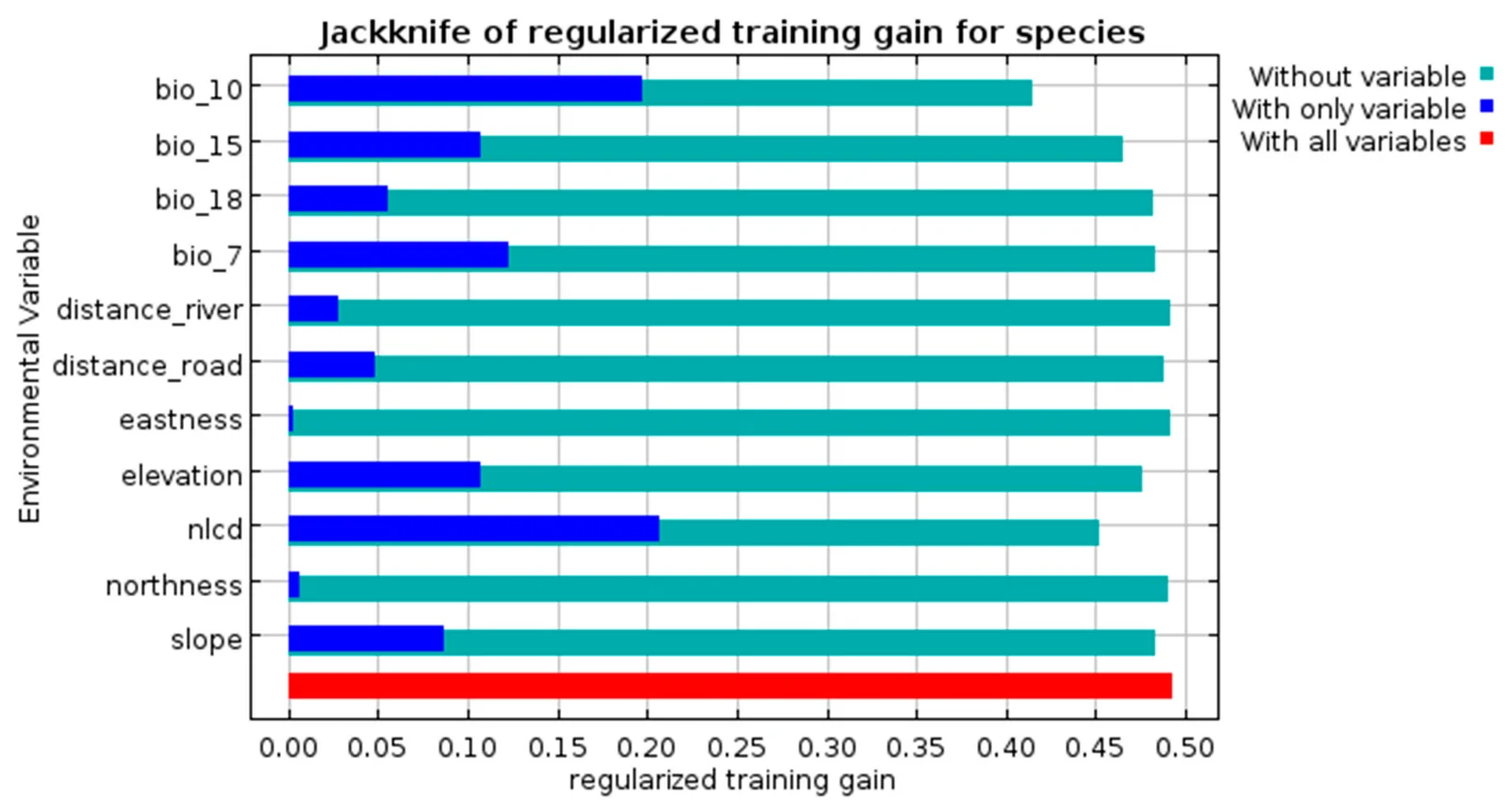
Jackknife test of environmental variable importance for the final MaxEnt model predicting Cerulean warbler habitat suitability. Blue bars show model gain when each predictor was used in isolation, turquoise bars show model gain when each predictor was omitted, and the red bar represents model gain using all predictors.

Environmental variable relative importance was evaluated using the percent contribution generated by the MaxEnt model, together with the Jackknife test results (Table 4; Figure A4). The final MaxEnt model identified land cover (NLCD) and temperature of the warmest quarter (BIO10) as the most influential predictors of Cerulean warbler habitat suitability (Table 4). NLCD contributed the largest proportion during model training (35.6%), followed by BIO10 (29.5%) and elevation (12.8%). However, permutation importance indicated that BIO10 was the most influential independent predictor (41.2%), followed by NLCD (15.2%), BIO15 (11.0%), and elevation (10.4%). The Jackknife test revealed that the environmental variable with the highest model performance when used in isolation is land cover. The environmental variable that causes the largest decline in the model performance when it is omitted is temperature of the warmest quarter, which therefore appears to have the most amount of unique information among all predictors.

- Variable response curves

**Figure A5:**
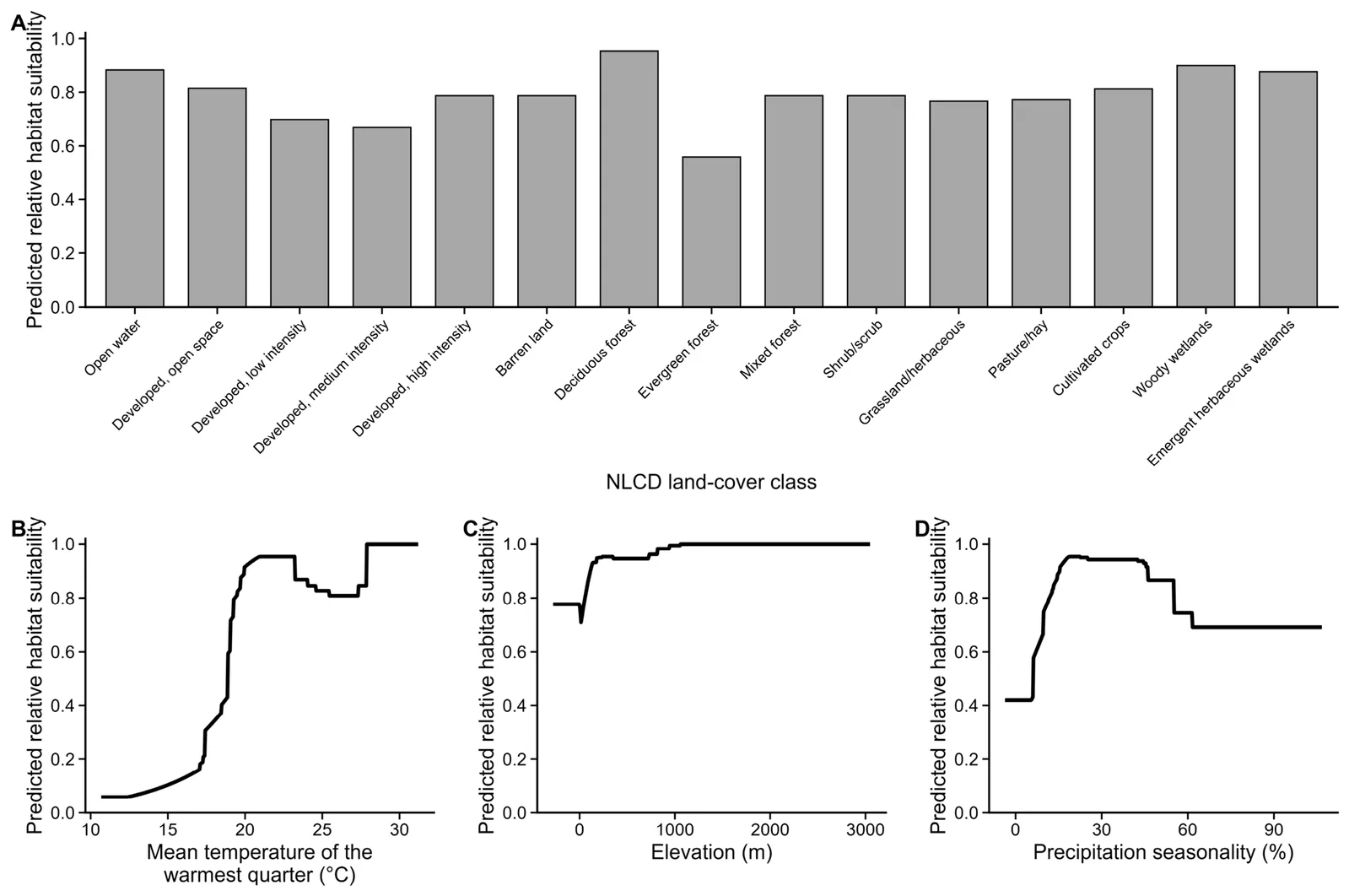
Marginal response plots for the four most influential predictors in the final MaxEnt model: (**A**) National Land Cover Database (NLCD) land-cover class, (**B**) mean temperature of the warmest quarter (BIO10), (**C**) elevation, and (**D**) precipitation seasonality (BIO15). Each curve shows how the predicted probability of presence changes as each environmental variable is varied, keeping all other environmental variables at their average sample value.

**Figure A6:**
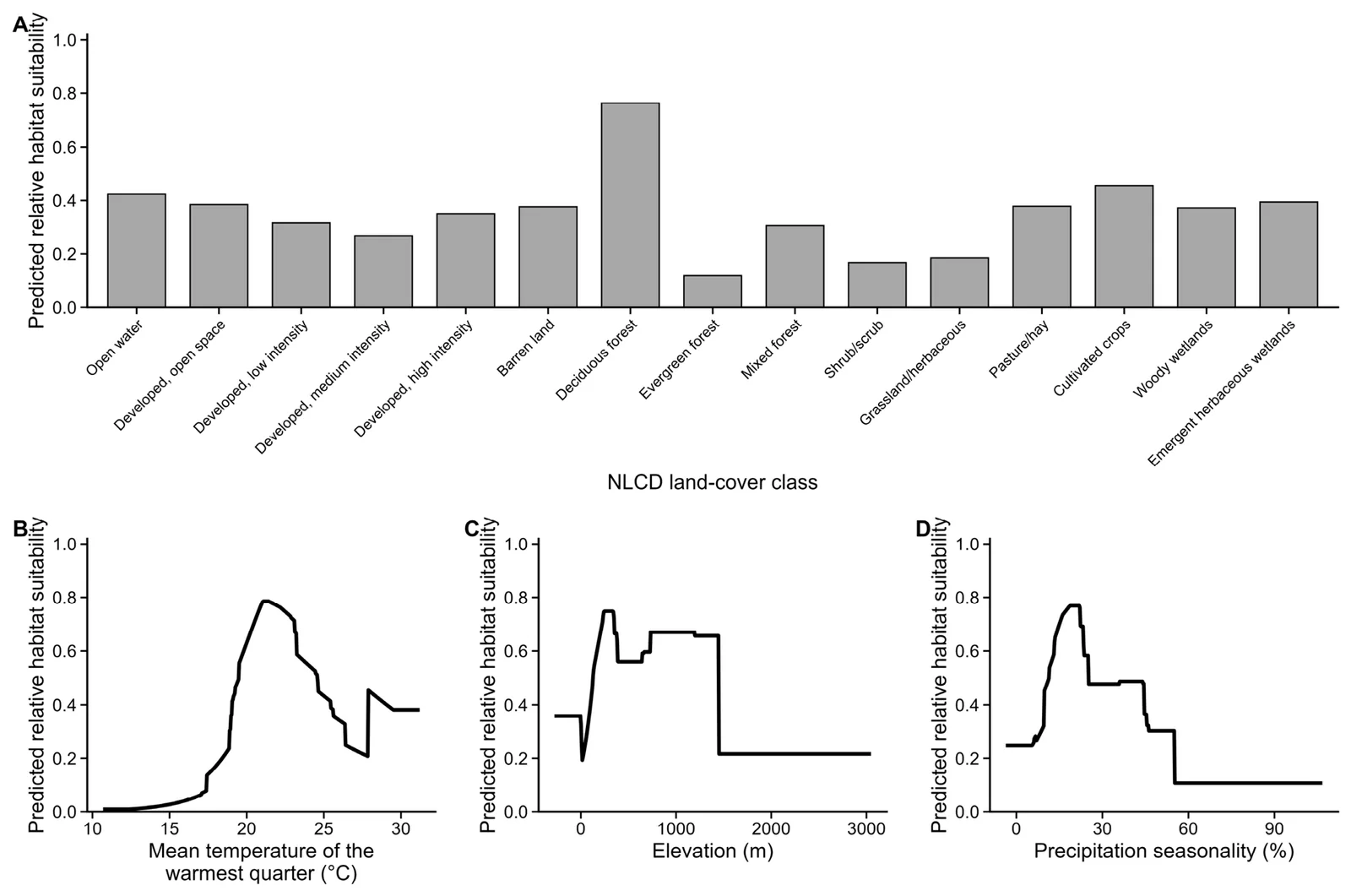
Single-variable response curve for the four most influential predictors in the final MaxEnt model: (**A**) National Land Cover Database (NLCD) land-cover class, (**B**) mean temperature of the warmest quarter (BIO10), (**C**) elevation, and (**D**) precipitation seasonality (BIO15). Each curve reflects the dependence of predicted suitability both on the selected variable and on dependencies induced by correlations between the selected variable and other variables.

The response curves generated by the MaxEnt model showed the relationship between each environmental predictor and predicted Cerulean warbler habitat suitability (Figure A4 and Figure A5). Marginal response curves, which show the response to each predictor while accounting for the other variables in the fitted model, sometimes differed from response curves generated from models containing each predictor individually. For example, the marginal response curves showed relatively high predicted suitability for deciduous forest as well as several nonforest land-cover classes, while suitability remained high across much of the upper elevation range (Figure A5). However, these patterns were less pronounced in the corresponding single-variable models (Figure A6). When land cover was modeled independently, deciduous forest had the highest predicted relative suitability, whereas most nonforest classes had lower predictions.

Differences were also evident for climatic and topographic predictors. The marginal response to mean temperature of the warmest quarter (BIO10) increased rapidly between approximately 18 and 20 °C and remained relatively high at warmer temperatures, with a modest decline between approximately 24 and 27 °C. In contrast, the single-variable response peaked at approximately 21–22 °C and declined more strongly at warmer temperatures. Similarly, the marginal elevation response increased rapidly to approximately 300 m and remained relatively high thereafter, whereas the single-variable response showed highest predicted suitability at low-to-intermediate elevations and declined substantially above approximately 1500 m. The single-variable response to precipitation seasonality was highest at relatively low-to-moderate values, peaking near 20% before declining at greater seasonality.

The single-variable response for mean temperature of the warmest quarter (BIO10) increased rapidly between approximately 18 and 21 °C, peaked near 21–22 °C, and declined at warmer temperatures. Similarly, the single-variable elevation response was highest at low-to-intermediate elevations and declined substantially above approximately 1500 m. Precipitation seasonality showed its highest predicted suitability at relatively low-to-moderate values, peaked near 20% before declining at higher seasonality. Differences between the marginal and single-variable curves indicate that the marginal responses were influenced by the multivariable structure of the model; therefore, these curves were interpreted as model responses rather than direct evidence of habitat preference.

The differences between marginal and single-variable response curves indicate that predicted responses to individual environmental variables depended partly on their relationships with other predictors in the multivariable model. Accordingly, the response curves were interpreted as descriptions of model behavior and predictor associations with predicted suitability, rather than as direct evidence of independent ecological preferences or thresholds.

### 3.2. Fine-Scale Habitat Association with Cerulean Warbler Acoustic Detection

The supplementary forest-change assessment indicated limited mapped tree-cover loss between LiDAR acquisition and acoustic sampling. Of the 57 unique sampling locations, 55 (96.5%) showed no mapped tree-cover loss from 2019–2025. Mapped loss occurred within the 50 m buffers of only two locations, affecting approximately 14.0% and 2.4% of their respective buffer areas.

Comparisons of habitat characteristics between acoustic sensor locations with and without confirmed Cerulean warbler detections indicated no statistically significant differences in the habitat variables evaluated at the primary 50 m spatial scale (all *p* > 0.05; Table 5). EVI showed the strongest tendency, with higher values at detection locations (0.65 ± 0.08) than at non-detection locations (0.59 ± 0.10; Cohen’s d = 0.60, *p* = 0.057). Vegetation cover within the 10–20 m height stratum also tended to be lower at detection locations [median = 0.19 (IQR: 0.14–0.25)] than at non-detection locations [0.23 (0.20–0.28); rank-biserial r = −0.32, *p* = 0.074].

Five candidate logistic regression models and an intercept-only null model were compared at the 50 m scale to evaluate relationships between confirmed Cerulean warbler acoustic detection and mean vegetation height (z_mean), 25th-percentile return height (z_p25), vegetation cover within the 5–10 m (cover_5_10) and 10–20 m (cover_10_20) height strata, and EVI. The EVI model was the highest-ranked candidate (AICc = 63.8, ΔAICc = 0, Akaike weight = 0.40). However, the 10–20 m vegetation-cover model (ΔAICc = 1.33, weight = 0.21) and the intercept-only null model (ΔAICc = 1.81, weight = 0.16) were also competitive. The remaining models received less support (ΔAICc ≥ 3.19; Table A5). Thus, model comparison indicated model-selection uncertainty and did not identify a single strongly supported fine-scale habitat predictor.

Results showed some sensitivity to the spatial scale used to characterize vegetation structure. In the 25 m sensitivity analysis, vegetation cover within the 5–10 m height stratum was significantly lower at detection locations [median = 0.05 (IQR: 0.04–0.09)] than at non-detection locations [0.09 (0.06–0.12); rank-biserial r = −0.41, *p* = 0.024]. The 5–10 m vegetation-cover model was also the highest-ranked candidate at the 25 m scale (AICc = 63.7, ΔAICc = 0, Akaike weight = 0.31). Nevertheless, the EVI model (ΔAICc = 0.54, weight = 0.24), 10–20 m vegetation-cover model (ΔAICc = 1.16, weight = 0.17), and intercept-only null model (ΔAICc = 1.92, weight = 0.12) were also competitive. These results indicate that model rankings were sensitive to the spatial scale used to summarize local habitat characteristics.

**Figure 2:**
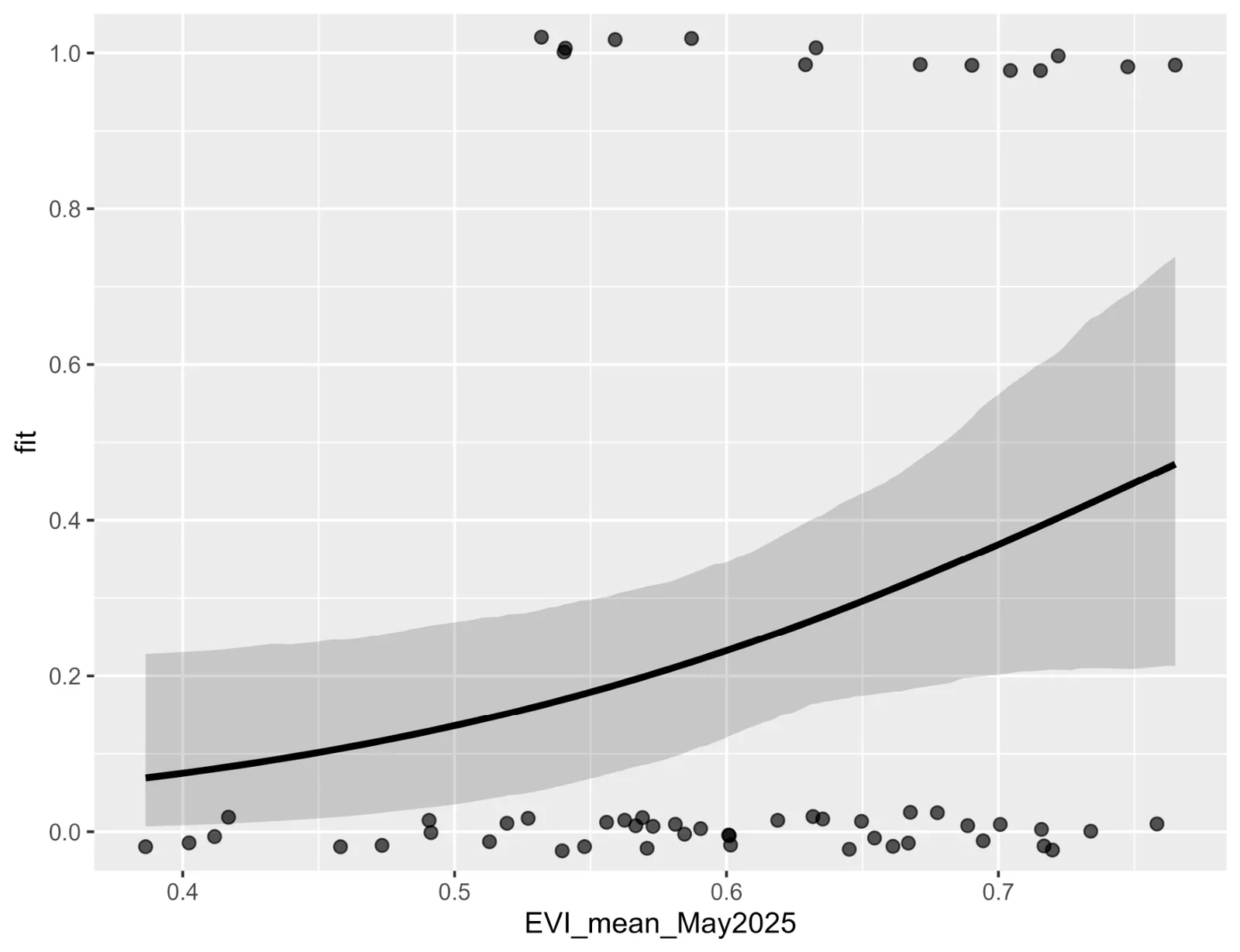
Firth-penalized logistic regression showing the relationship between EVI and the probability of Cerulean warbler detection at 50 m buffer. The solid line represents the fitted model, the shaded region indicates the 95% confidence interval, and points represent observed detection (1) and non-detection (0) records.

**Figure A7:**
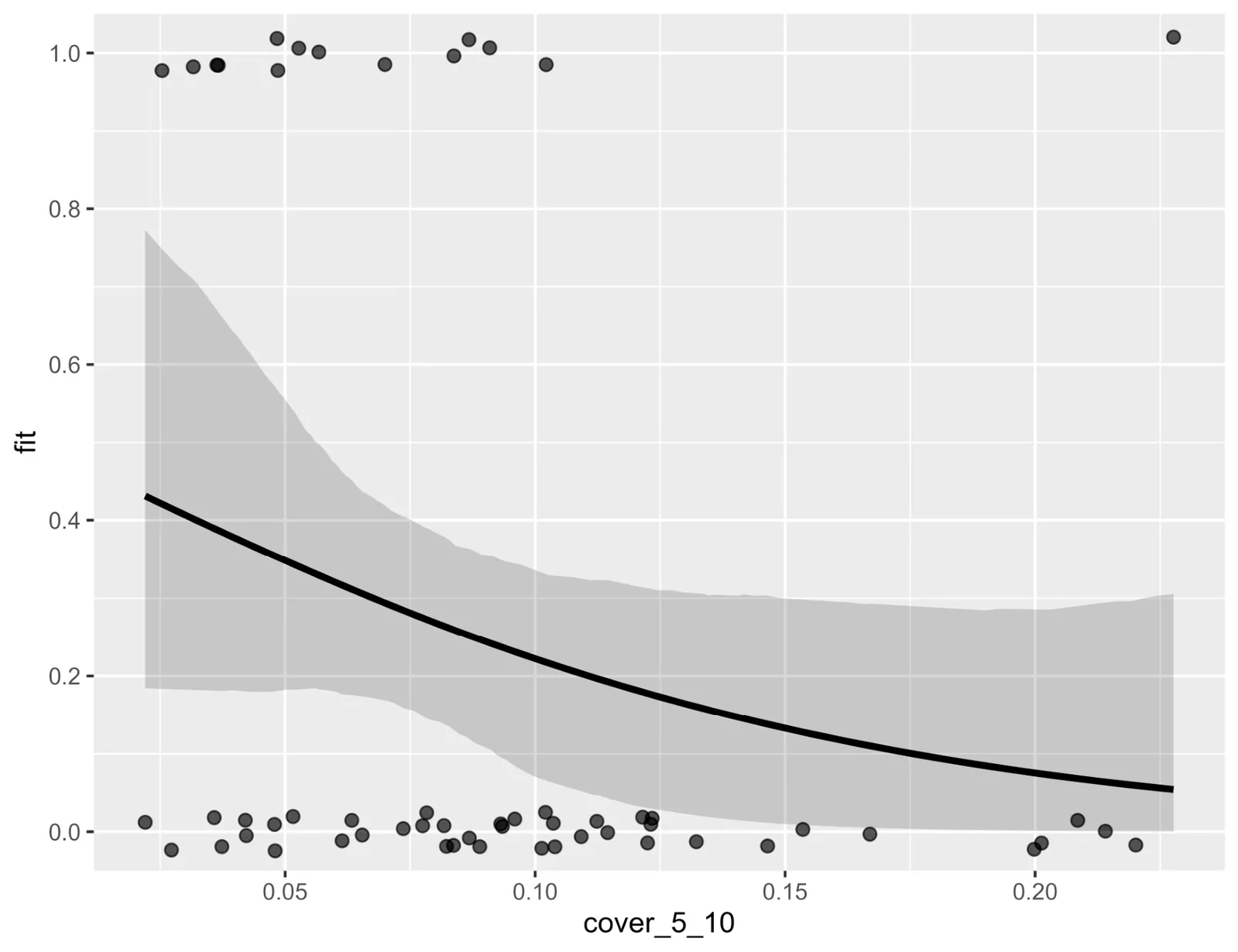
Firth-penalized logistic regression showing the relationship between vegetation cover within the 5–10 m height stratum and the probability of Cerulean warbler detection at 50 m buffer. The solid line represents the fitted model, the shaded region indicates the 95% confidence interval, and points represent observed detection (1) and non-detection (0) records.

Firth-penalized logistic regression indicated scale-dependent but statistically uncertain fine-scale habitat associations. At the 25 m scale, vegetation cover within the 5–10 m height stratum was negatively associated with confirmed Cerulean warbler acoustic detection (β = −12.50, 95% CI: −29.74 to 0.64, *p* = 0.064) Figure A7. Expressed as an odds ratio, each 10-percentage-point increase in 5–10 m vegetation cover was associated with lower odds of detection (OR = 0.29, 95% CI: 0.05–1.07), although the confidence interval included 1. At the 50 m scale, EVI showed a positive association with acoustic detection (β = 6.56, 95% CI: −0.21 to 14.50, *p* = 0.058) Figure 2. A 0.10-unit increase in EVI corresponded to an estimated 1.93-fold increase in the odds of detection (OR = 1.93, 95% CI: 0.98–4.26), although this association also did not reach statistical significance.

Sensitivity analyses showed that the direction of the focal associations was generally consistent across reserves, but statistical precision was limited. At the 25 m scale, coefficients for 5–10 m vegetation cover remained negative in all leave-one-reserve-out analyses (β = −29.61 to −7.89), although confidence intervals overlapped zero in three of the four analyses. At the 50 m scale, EVI coefficients remained positive regardless of which reserve was excluded (β = 5.58–9.28), but confidence intervals overlapped zero in three of the four analyses. Reserve-adjusted Firth models likewise retained the same direction of effect at both scales, although neither association was statistically significant after adjustment (25 m: β = −10.93, *p* = 0.132; 50 m: β = 9.48, *p* = 0.066). Residual spatial autocorrelation was not statistically significant at either scale (25 m: Moran’s I = 0.037, permutation *p* = 0.225; 50 m: Moran’s I = 0.086, permutation *p* = 0.120), providing no evidence of residual positive spatial clustering under the four-nearest-neighbor spatial weights structure. These results indicate that the observed fine-scale habitat associations were directionally consistent but sensitive to spatial scale and limited by uncertainty associated with the small number of confirmed detections.

## 4. Discussion

Understanding habitat requirements is fundamental for the conservation of declining forest songbirds such as the Cerulean warbler. This study integrated a multi-sensor ecological monitoring pipeline to characterize habitat requirements of the Cerulean warbler. A MaxEnt model was developed to predict habitat suitability across the contiguous United States and exhibited moderate predictive performance. At a finer spatial scale, passive acoustic monitoring combined with LiDAR-derived structural metrics and satellite remote sensing was used to characterize forest structural conditions associated with Cerulean warbler detections. This integrated sensor-based workflow highlights the potential of combining passive acoustic monitoring, remote sensing, and machine-learning approaches for biodiversity monitoring and conservation planning.

### 4.1. Broad-Scale Habitat Suitability Patterns

The MaxEnt model identified substantial geographic variation in habitat suitability across the contiguous United States, with areas of highly suitable habitats concentrated primarily in the eastern United States. These results are consistent with previous studies, which have reported Cerulean warbler occurrences in areas such as eastern Kentucky [47], southern West Virginia [7,26], Tennessee [48], Appalachian Mountains region [49,50], Indiana [51,52]. The predictive performance of the model was moderate based on the mean validation AUC of 0.760 under spatial block cross-validation. MaxEnt has been widely used for modeling species distributions using presence-only data and has demonstrated strong performance across taxa [38,53,54]. Land cover (NLCD) contributed the most to model training (35.6% contribution). Studies have also found effects of land cover change on avian species richness, abundance, distribution, and population trends of forest songbird species [55,56,57,58]. The NLCD response curve indicated that deciduous forest land cover provided the highest predicted suitability for the species, consistent with prior findings that Cerulean warblers primarily occupy mature deciduous forests [48]. Deciduous forests provide large canopy trees, structurally complex vegetation, and abundant arthropod prey [12,59], all of which are important for nesting and foraging. Mean temperature of the warmest quarter (BIO10) was the most influential independent predictor (41.2% permutation importance), suggesting that breeding-season temperature is an important climatic variable of Cerulean warbler habitat suitability. Breeding-season temperatures can influence vegetation phenology, insect emergence, and breeding conditions, thereby indirectly affecting habitat suitability for insectivorous migratory birds [60]. Elevation was the third most important predictor, with predicted habitat suitability increasing rapidly up to approximately 300 m and remaining high thereafter. Previous studies have likewise identified elevation as an important determinant of Cerulean warbler occurrence, although the direction of the relationship has varied among the studies [24,61]. For example, studies conducted in Appalachian and southern Indiana forests reported greater occupancy at lower elevations [24,61], whereas a habitat-selection study in the Cumberland Mountains of Tennessee found that Cerulean warblers preferentially selected higher-elevation sites located on ridge tops and upper slopes [48].

### 4.2. Fine-Scale Habitat Characteristics

Fine-scale habitat associations with Cerulean Warbler detections varied with the spatial scale at which vegetation characteristics were summarized. At the 25 m scale, vegetation cover within the 5–10 m height stratum was significantly lower at detection than non-detection sites in the exploratory univariate comparison and was the highest-ranked predictor in the candidate model set. The direction of this relationship suggests that Cerulean warbler detections tended to occur at locations with relatively open midstory vegetation, consistent with previous studies describing Cerulean warbler habitat as mature forest with relatively open midstory structure and well-developed upper canopies [8,62]. Similar relationships between canopy closure, vertical structure, and avian habitat use have been documented in forest bird studies using LiDAR-derived metrics [25,63]. Habitat structural heterogeneity can influence the distribution of resources, foraging opportunities, and niche partitioning within forest ecosystems [64,65]. However, support for the 5–10 m vegetation-cover relationship was not conclusive. Several candidate models received comparable AICc support, and the negative association in the Firth-penalized logistic regression was imprecisely estimated and did not reach statistical significance. Thus, lower vegetation cover within the 5–10 m stratum should be interpreted as a potential local habitat association rather than evidence of a definitive structural preference.

At the broader 50 m scale, EVI rather than a LiDAR-derived structural metric was the highest-ranked predictor, with detection sites tending to have higher EVI values. Higher EVI generally reflects greater photosynthetically active vegetation and vegetation greenness and can therefore provide a measure of vegetation productivity [66]. This positive association may indicate that Cerulean warbler detections occurred in areas with greater vegetation productivity at the 50 m scale. Greater vegetation productivity may influence habitat conditions relevant to insectivorous birds, including food-resource availability and vegetation characteristics [67]. However, the EVI relationship was not statistically significant, and several other models had similar support. Therefore, the EVI pattern should be interpreted cautiously.

Previous studies have reported that Cerulean warblers are widely known as canopy specialists that occupy mature forest with tall trees, complex vertical structure, and well-developed upper canopy layers [4,8]. These forest characteristics have been hypothesized to provide important ecological benefits for canopy-dwelling songbirds, including increased foraging opportunities [68], reduced nest predation risk, and the availability of suitable nesting substrates and microclimatic stability for incubating females [69]. In this study, Cerulean warbler detections tended to occur at sites with lower 5–10 m vegetation cover within 25 m and higher EVI within 50 m. This suggests that local vegetation structure and broader vegetation conditions may be associated with detections at different spatial scales. However, because several models received similar support, further studies are needed to confirm these relationships.

### 4.3. Limitations and Future Directions

This study focused on the occurrence of Cerulean warbler acoustic detections across different forest types rather than measures of abundance, occupancy, or vocal activity levels. Because passive acoustic monitoring provides detection/non-detection data rather than direct counts of individuals, the observed relationships should be interpreted as associations with acoustic detection rather than definitive evidence of habitat selection or population size. In addition, detection probability may vary with vegetation structure, as a more open midstory could improve sound propagation and increase the likelihood that BirdNET detects and correctly classifies vocalizations. Detection probability may also be influenced by recorder model (SM3 and SM4), recording effort, deployment period, weather conditions, background noise, and time of day, none of which were explicitly modeled in the present study. Consequently, the observed negative association between Cerulean warbler acoustic detections and vegetation cover within the 5–10 m stratum should be interpreted with caution, as it may reflect both ecological patterns and habitat-dependent detectability. Future research should incorporate occupancy or repeated-detection modeling frameworks that explicitly account for imperfect detection, together with measures of call rate, detection frequency, or abundance, to provide more robust ecological inference.

The fine-scale component of this study was restricted to a single month during the breeding season. Although May corresponds to peak arrival and territorial activity for Cerulean warblers in the Midwest, habitat use may vary across the breeding period and throughout migration. Longer-term deployments spanning the full breeding season and migratory windows would allow examination of temporal dynamics in habitat use, vocal activity patterns, and phenology, including arrival and departure timing. Multi-season monitoring would also help distinguish transient migrants from breeding individuals.

At the broad spatial scale, the species distribution model relied primarily on citizen science occurrence data. While such datasets provide extensive spatial coverage, they may contain sampling biases related to observer effort, accessibility, and detectability [70,71]. Future work could improve model reliability by integrating citizen science records with standardized survey data or ground-validated acoustic observations.

Another limitation of this study is the temporal mismatch between the LiDAR acquisition and the passive acoustic surveys conducted during the 2025 breeding season. A supplementary assessment using the Hansen Global Forest Change dataset indicated limited major canopy disturbance during this period, with 55 of 57 sampling locations showing no mapped tree-cover loss from 2019–2025. However, the absence of mapped tree-cover loss does not demonstrate that vertical forest structure remained unchanged. Therefore, the fine-scale habitat relationships should still be interpreted cautiously. Nevertheless, future studies should prioritize temporally aligned LiDAR acquisition and acoustic sampling to minimize uncertainty associated with temporal mismatches.

An additional limitation of this study was the relatively small number of Cerulean warbler detection sites (n = 14), which limited statistical power and the complexity of models that could be fitted. Consequently, the analyses focused on parsimonious models evaluating individual habitat variables rather than multivariable models incorporating many predictors. Also, BirdNET detections were manually validated, and a confidence threshold of 0.40 was selected to minimize false positives; sites without detections cannot be interpreted as confirmed absences because individuals may have been present but undetected during the recording period. Consequently, the fine-scale analyses compared sites with confirmed detections to sites without confirmed detections rather than true presence–absence locations. Future studies incorporating occupancy modeling or repeated-detection frameworks could account for imperfect detection.

Finally, although habitat characteristics were evaluated within 25 m and 50 m buffers, these scales primarily represent local vegetation conditions surrounding the acoustic sensors. Cerulean warbler occurrence may also be influenced by habitat characteristics operating at broader spatial scales. Future studies should therefore evaluate local vegetation structure together with landscape-level factors, such as forest cover, patch size, and fragmentation, to better understand how habitat associations change across spatial scales and inform conservation and forest management of this Near-Threatened species.

## 5. Conclusions

In this study, we integrated passive acoustic sensors, LiDAR, and satellite remote sensing for habitat assessment of the Cerulean warbler. A MaxEnt approach was used to identify the extent and geographic distribution of suitable habitat for the Cerulean warbler across the contiguous United States, highlighting the importance of land cover, climatic conditions, and elevation in shaping large-scale habitat suitability. The model results indicate that suitable habitat is unevenly distributed, emphasizing the need for targeted conservation efforts in regions that provide the best environmental conditions for the species. The fine-scale assessment, which complemented the broad-scale analysis, integrated passive acoustic detections, LiDAR-derived forest structural metrics, and Sentinel-2 vegetation indices to provide detailed insights into habitat characteristics at acoustic detection locations in west-central Indiana. Habitat associations differed with the spatial scale considered. At 25 m, lower vegetation cover within the 5–10 m height stratum received the greatest model support, whereas at 50 m, higher EVI received the greatest support. However, several models were competitive at both scales, indicating uncertainty in identifying a single dominant fine-scale habitat predictor. Overall, this study demonstrates how broad-scale habitat suitability modeling and fine-scale habitat characterization provide complementary perspectives on Cerulean warbler habitat requirements. While MaxEnt identified potential breeding habitat across the contiguous United States, passive acoustic monitoring combined with LiDAR and Sentinel-2 data characterized local habitat conditions associated with species detection. Together, these approaches provide valuable information for conservation planning and habitat management of the Cerulean warbler.

## Figures and Tables

**Table 1:** Summary of data sources used in this study.

Data	Source
Occurrence record of Cerulean warbler	Global Biodiversity Information Facility (GBIF) https://www.gbif.org/ and downloaded on 12 September 2025 (GBIF Occurrence Download, https://doi.org/10.15468/dl.zkdfx7
Nineteen bioclimatic variables	WorldClim database. (http://www.worldclim.org/, accessed on 21 September 2024)
Elevation data	U.S. Geological Survey (USGS) 3D Elevation Program (https://www.usgs.gov/3d-elevation-program)
Land cover data	National Land Cover Database (NLCD) (https://www.mrlc.gov/data)
Rivers and roads shapefile	United States Census Bureau TIGER/Line dataset (https://www.census.gov/geographies/mapping-files/time-series/geo/tiger-line-file.html)
LiDAR data	(USGS 3DEP/local LiDAR repositories depending on study area)
Sentinel imagery	https://dataspace.copernicus.eu/data-collections/copernicus-sentinel-missions/sentinel-2

**Table 2:** Environmental predictor variables considered for MaxEnt modeling of Cerulean warbler habitat suitability, including variable units, original spatial resolution, resampling or aggregation methods, results of correlation and variance inflation factor (VIF) screening, final VIF values, and inclusion in the final model.

Variable	Unit	Original Resolution	Resampling/Aggregation	Correlation Screening	VIF Screening	Final VIF	Included in Final Model
BIO1	°C	30 arc-sec (~1 km)	None	Removed	Removed	—	No
BIO2	°C	30 arc-sec	None	Removed	Removed	—	No
BIO3	%	30 arc-sec	None	Removed	Removed	—	No
BIO4	SD × 100	30 arc-sec	None	Removed	Removed	—	No
BIO5	°C	30 arc-sec	None	Removed	Removed	—	No
BIO6	°C	30 arc-sec	None	Removed	Removed	—	No
BIO7	°C	30 arc-sec	None	Retained	Retained	1.4	Yes
BIO8	°C	30 arc-sec	None	Removed	Removed	—	No
BIO9	°C	30 arc-sec	None	Removed	Removed	—	No
BIO10	°C	30 arc-sec	None	Retained	Retained	3.1	Yes
BIO11	°C	30 arc-sec	None	Removed	Removed	—	No
BIO12–14	mm	30 arc-sec	None	Removed	Removed	—	No
BIO15	mm	30 arc-sec	None	Retained	Retained	1.9	Yes
BIO16–17	mm	30 arc-sec	None	Removed	Removed	—	No
BIO18	mm	30 arc-sec	None	Retained	Retained	2.2	Yes
BIO19	mm	30 arc-sec	None	Removed	Removed	—	No
Elevation	m	30 m	Bilinear	Retained	Retained	2.4	Yes
Slope	degrees	Derived from DEM	Bilinear	Retained	Retained	1.6	Yes
Aspect	degrees	Derived from DEM	Not used directly	—	—	—	No
Northness	unitless	Derived from aspect	Bilinear	Retained	Retained	1.0	Yes
Eastness	unitless	Derived from aspect	Bilinear	Retained	Retained	1.0	Yes
Distance to road	m	Vector (TIGER roads)	Euclidean distance raster → Bilinear	Retained	Retained	1.0	Yes
Distance to river	m	Vector (river network)	Euclidean distance raster → Bilinear	Retained	Retained	1.2	Yes
NLCD	Category	30 m	Majority (mode) aggregation to 1 km	Retained	Retained	—	Yes

**Table 3:** Spatial block cross-validation performance of the selected MaxEnt model.

Model	Spatial Fold	Validation AUC	AUC Difference	CBI	MTP Omission	10% Omission
LQHPT, RM = 1	1	0.703	0.15	0.74	0.002	0.32
LQHPT, RM = 1	2	0.835	0.02	0.97	0.002	0.14
LQHPT, RM = 1	3	0.723	0.11	0.91	0.005	0.30
LQHPT, RM = 1	4	0.780	0.05	0.98	0.000	0.16

Abbreviations: L = linear, Q = quadratic, H = hinge, P = product, and T = threshold feature classes; RM = regularization multiplier; AUC = area under the receiver operating characteristic curve; CBI = continuous Boyce index; MTP = minimum training presence.

**Table 4:** Environmental variable contributions of derived MaxEnt model.

Variable	Percent Contribution	Permutation Importance
nlcd	35.6	15.2
bio_10	29.5	41.2
elevation	12.8	10.4
bio_15	7.8	11
bio_7	5.8	7.3
bio_18	3.2	6.4
slope	2.4	4.4
distance_road	1.6	1.9
northness	0.6	1.2
distance_river	0.5	0.8
eastness	0.2	0.3

**Table 5:** Exploratory univariate comparisons of LiDAR-derived habitat variables and Enhanced Vegetation Index (EVI) between sensor locations with confirmed Cerulean warbler acoustic detections (n = 14) and non-detections (n = 43). Variables were compared using Student’s *t*-tests when assumptions of normality were met, and Mann–Whitney U tests otherwise. Values are presented as mean ± SD for *t*-tests and median (interquartile range) for Mann–Whitney U tests. Effect sizes are reported as Cohen’s d for *t*-tests and rank-biserial correlation (r) for Mann–Whitney U tests.

Variable	Detection (n = 14)	Non-Detection (n = 43)	Test	Effect Size	*p*
Mean canopy height (m)	9.48 Â ± 3.71	8.56 Â ± 3.85	Student’s *t*	d = 0.24	0.44
Maximum canopy height (m)	31.29 (28.32–35.88)	29.68 (25.75–32.34)	Mann–Whitney	r = 0.24	0.185
Canopy height SD (m)	10.18 (8.58–10.62)	8.98 (7.23–10.27)	Mann–Whitney	r = 0.24	0.185
25th height percentile (m)	0.05 (0.03–0.07)	0.03 (0.02–0.08)	Mann–Whitney	r = 0.15	0.404
50th height percentile (m)	9.14 (3.50–12.91)	7.18 (2.16–9.75)	Mann–Whitney	r = 0.15	0.409
75th height percentile (m)	20.76 (15.71–21.77)	17.01 (10.94–20.78)	Mann–Whitney	r = 0.21	0.254
95th height percentile (m)	26.56 (23.09–27.04)	22.87 (19.93–27.18)	Mann–Whitney	r = 0.21	0.247
Vegetation cover (>2 m)	0.59 (0.54–0.61)	0.58 (0.51–0.61)	Mann–Whitney	r = 0.05	0.774
Vegetation cover (>5 m)	0.55 (0.47–0.58)	0.53 (0.42–0.58)	Mann–Whitney	r = 0.03	0.86
Vegetation cover (0–5 m)	0.42 (0.39–0.47)	0.43 (0.38–0.54)	Mann–Whitney	r = 0.03	0.875
Vegetation cover (5–10 m)	0.06 (0.05–0.10)	0.09 (0.06–0.10)	Mann–Whitney	r = −0.24	0.191
Vegetation cover (10–20 m)	0.19 (0.14–0.25)	0.23 (0.20–0.28)	Mann–Whitney	r = −0.32	0.074
Vegetation cover (>20 m)	0.28 (0.12–0.31)	0.18 (0.05–0.28)	Mann–Whitney	r = 0.22	0.224
Foliage height diversity	1.54 (1.40–1.63)	1.46 (1.21–1.60)	Mann–Whitney	r = 0.13	0.487
Enhanced Vegetation Index (EVI)	0.65 Â ± 0.08	0.59 Â ± 0.10	Student’s *t*	d = 0.60	0.057

Abbreviations: Values are presented as mean ± SD for variables analyzed using Student’s *t*-tests and as median (interquartile range) for variables analyzed using Mann–Whitney U tests. Cohen’s d is reported for *t*-tests, whereas rank-biserial correlation (r) is reported for Mann–Whitney U tests.

## Data Availability

The processed datasets and R scripts supporting the findings of this study have been deposited in a Figshare repository and will be made publicly available upon publication at https://figshare.com/s/6f518890b57a23f579af.

## Supplementary Materials

The following supporting information can be downloaded at: https://www.mdpi.com/article/10.3390/s26165152/s1, Table S1: ENMeval tuning results for all candidate MaxEnt models evaluated across combinations of feature classes (FC) and regularization multipliers (RM); Table S2: Spatial block cross-validation results for all candidate MaxEnt models evaluated using ENMeval.

## Appendix A

### Appendix A.1

**Table A1:** Summary of BirdNET detections and manual validation used to determine the species-specific confidence threshold for Cerulean warbler detections. The table reports the total number of BirdNET detections, the number of manually validated detections, true positives, false positives, and validation metrics used to select the final confidence threshold for subsequent analyses.

ConfidenceBin	Total_Detections	Reviewed	True_Detections	False_Detections	Precision (TP/(TP + FP))
[0.05, 0.1)	22,031	20	4	16	0.2
[0.1, 0.15)	4270	20	4	16	0.2
[0.15, 0.2)	1222	10	2	8	0.2
[0.2, 0.25)	437	10	6	4	0.6
[0.25, 0.3)	192	10	3	7	0.3
[0.3, 0.35)	96	10	5	5	0.5
[0.35, 0.4)	50	10	4	6	0.4
[0.4, 0.45)	31	10	9	1	0.9
[0.45, 0.5)	20	10	10	0	1
[0.5, 0.55)	8	8	7	1	0.875
[0.55, 0.6)	12	10	9	1	0.9
[0.6, 0.65)	3	3	3	0	1
[0.65, 0.7)	3	3	3	0	1
[0.7, 0.75)	2	2	1	1	0.5
[0.75, 0.8)	1	1	1	0	1
[0.9, 0.95)	1	1	1	0	1

### Appendix A.2

**Table A2:** Summary of site-level manual validation of Cerulean warbler detections across survey periods. For each positive sensor location, the table reports the number of BirdNET candidate detections (confidence ≥ 0.40), the number of recordings manually reviewed, and the number of manually confirmed Cerulean warbler detections.

ID	Survey	Site	Sensor	Candidate Detections	Clips Manually Reviewed	Confirmed Cerulean Warbler Detections
Cun_01	14th_May	Cunningham	S4A06603	1	1	1
Cun_02	26th_May	Cunningham	S4A06603	2	2	2
Cun_03	14th_May	Cunningham	S4A17591	1	1	1
Cun_04	26th_May	Cunningham	S4A17643	1	1	1
Mar_01	9th_May	Martell	MOT3-2	1	1	1
Mar_02	19th_May	Martell	S4A17581	6	3	3
PWA_01	19th_May	PWA	MOT2-1	1	1	1
PWA_02	9th_May	PWA	MOT3-6	1	1	1
PWA_03	19th_May	PWA	S4A03853	1	1	1
PWA_04	26th_May	PWA	S4A17590	2	2	2
Ross_01	26th_May	Ross	MOT3-4	1	1	1
Ross_02	19th_May	Ross	S4A00449	1	1	1
Ross_03	26th_May	Ross	S4A00449	4	3	3
Ross_04	26th_May	Ross	S4A00450	24	2	2

**Table A3:** Distribution of Cerulean warbler acoustic detection and non-detection locations among the four forest types.

Vegetation_Type	Non-Detection	Detection
mature deciduous forest	6	4
mixed forest_grassland mosaic	15	4
mixed hardwood forest	12	4
secondary managed forest	10	2

### Appendix A.3

**Table A4:** Leave-one-forest-type-out sensitivity analysis of the association between (a) EVI and Cerulean warbler acoustic detection at the 50 m spatial scale and (b) vegetation cover at 5–10 m stratum at the 25 m spatial scale.

**(a)**	**a. EVI at 50 m Scale**							
	**Forest Type Removed**	**N**	**Detections**	**Non_Detections**	**Beta**	**CI_Low**	**CI_High**	***p*** **_Value**
	mature deciduous forest	47	10	37	5.58	−2.60	15.10	0.19
	Mixed forest_grassland mosaic	38	10	28	5.78	−1.76	14.69	0.14
	mixed hardwood forest	41	10	31	9.28	0.13	22.16	0.05
	Secondary managed forest	45	12	33	6.21	−1.02	14.60	0.09
**(b)**	**b. Vegetation cover 5_10 m at 25 m scale**							
	**Forest type removed**	**N**	**Detections**	**Non_detections**	**Beta**	**CI_low**	**CI_high**	***p*** **_value**
	mature deciduous forest	47	10	37	−7.89	−24.92	5.45	0.26
	Mixed forest_grassland mosaic	38	10	28	−29.61	−62.65	−5.89	0.01
	mixed hardwood forest	41	10	31	−9.59	−27.68	3.67	0.17
	Secondary managed forest	45	12	33	−10.26	−28.62	3.52	0.16

### Appendix A.4

**Table A5:** Candidate generalized linear models evaluating the influence of forest structural and vegetation variables on Cerulean warbler detection. Models were ranked using Akaike’s Information Criterion corrected for small sample size (AICc). Shown are model coefficients, degrees of freedom (df), log-likelihood (logLik), AICc, ΔAICc, and Akaike weight. The model with the lowest AICc was considered the best-supported model.

Model	(Intercept)	EVI_Mean	Cover_10_20	z_p25	z_Mean	Cover_5_10	df	logLik	AICc	Delta	Weight
Enhanced Vegetation Index (EVI)	−5.54	7.13	NA	NA	NA	NA	2	−29.80	63.82	0.00	0.40
Vegetation cover (10–20 m)	0.10	NA	−5.83	NA	NA	NA	2	−30.46	65.14	1.33	0.21
Null (intercept-only)	−1.12	NA	NA	NA	NA	NA	1	−31.78	65.62	1.81	0.16
25th height percentile	−1.01	NA	NA	−1.59	NA	NA	2	−31.39	67.01	3.19	0.08
Mean canopy height	−1.72	NA	NA	NA	0.07	NA	2	−31.46	67.14	3.32	0.08
Vegetation cover (5–10 m)	−0.68	NA	NA	NA	NA	−5.25	2	−31.57	67.35	3.54	0.07

Note: NA = not applicable; the predictor was not included in the corresponding model.
